# A Jurkat 76 based triple parameter reporter system to evaluate TCR functions and adoptive T cell strategies

**DOI:** 10.18632/oncotarget.24807

**Published:** 2018-04-03

**Authors:** Sandra Rosskopf, Judith Leitner, Wolfgang Paster, Laura T. Morton, Renate S. Hagedoorn, Peter Steinberger, Mirjam H.M. Heemskerk

**Affiliations:** ^1^ Institute of Immunology, Center for Pathophysiology, Infectiology and Immunology, Medical University of Vienna, Vienna, Austria; ^2^ Department of Hematology, Leiden University Medical Center, Leiden, The Netherlands

**Keywords:** adoptive T cell therapy, TCR gene transfer, immunotherapy, reporter T cells, chimeric receptors

## Abstract

Adoptive T cell therapy using TCR transgenic autologous T cells has shown great potential for the treatment of tumor patients. Thorough characterization of genetically reprogrammed T cells is necessary to optimize treatment success. Here, we describe the generation of triple parameter reporter T cells based on the Jurkat 76 T cell line for the evaluation of TCR and chimeric antigen receptor functions as well as adoptive T cell strategies. This Jurkat subline is devoid of endogenous TCR alpha and TCR beta chains, thereby circumventing the problem of TCR miss-pairing and unexpected specificities. The resultant reporter cells allow simultaneous determination of the activity of the transcription factors NF-κB, NFAT and AP-1 that play key roles in T cell activation. Human TCRs directed against tumor and virus antigens were introduced and reporter responses were determined using tumor cell lines endogenously expressing the antigens of interest or via addition of antigenic peptides. Finally, we demonstrate that coexpression of adhesion molecules like CD2 and CD226 as well as CD28 chimeric receptors represents an effective strategy to augment the response of TCR-transgenic reporters to cells presenting cognate antigens.

## INTRODUCTION

Adoptive transfer of T cell receptor-transgenic (TCR-tg) autologous T cells holds great promise for the treatment of tumor patients [1, 2]. Extensive pre-clinical evaluation is required to gauge the therapeutic potential of TCR molecules. Such studies often rely on T cell clones harboring the TCR of interest as well as primary human TCR-tg T cells. The human Jurkat T cell line has also served as a useful model to study TCRs specific for HLA class I restricted tumor and virus antigens as well as MHC class II restricted antigens [3–6]. Mispairing of introduced TCR chains with endogenous TCR chains is a major concern for genetic approaches aimed at re-directing human T cells to tumors or virus infected cells. This could potentially result in autoreactive T cells and will greatly reduce the TCR complexes with the desired specificity on the surface of the engineered T cells or T cell lines [7, 8].

J76, a Jurkat E6.1 subline that is devoid of endogenous TCR alpha and TCR beta chains, has been generated to study the response of TCR-tg T cells without the problem of TCR mispairing [9]. These cells have been widely used to study TCRs recognizing a variety of antigens presented by different HLA class I and class II molecules as well as non-classical HLA molecules [7, 10–14]. We have recently described the generation of a fluorescent triple parameter transcriptional reporter T cell line that can be used to simultaneously measure the activity of the transcription factors nuclear factor kappa-light-chain-enhancer of activated B-cells (NF-κB), nuclear factor of activated T cells (NFAT), and transcription factor activator protein-1 (AP-1), which play major roles in T cell activation processes [15]. These triple parameter reporter (short designation TPR) cells were shown to be a useful tool to evaluate signals transduced upon engagement of the TCR-complex and also accessory molecules like costimulatory and coinhibitory receptors [16].

Here we describe the generation of TPR cells based on the Jurkat 76 T cell line (J76). These cells were engineered to express selected human TCRs directed against tumor and virus antigens. Tumor cell lines endogenously expressing the antigens of interest and peptide loaded cells were used to test the antigen-specific responses of TCR-tg J76 TPR cells. In addition, we have introduced adhesion molecules and chimeric receptors in our TCR-tg reporter cells to study the capacity of these molecules to increase antigen-specific responses. Taken together, our results show that TCR-tg TPRs are useful tools to evaluate T cell responses to tumor and virus antigens. In addition, they allow to readily evaluate strategies to enhance responses of engineered T cells to tumor cells by introducing additional activating signals.

## RESULTS

### Generation and characterization of Jurkat J76-based triple parameter reporter T cells

**Figure 1:**
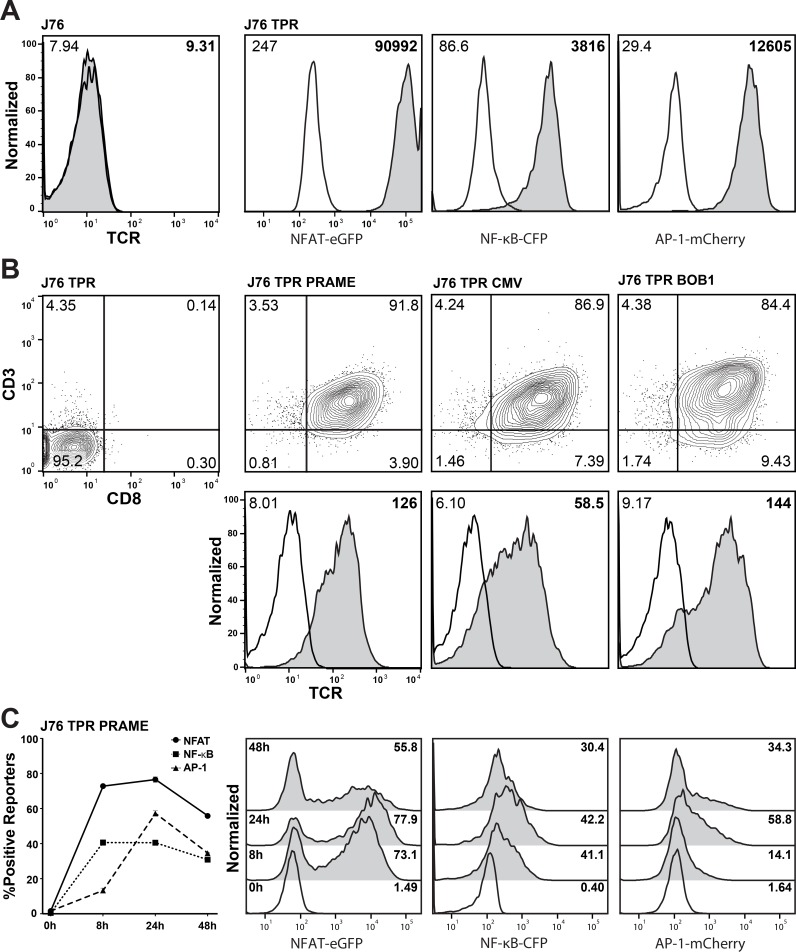
Generation of a TCR-transgenic triple parameter reporter T cell line (**A**) TCR-αβ-negative Jurkat 76 cells were transduced with three reporter constructs (NFAT-eGFP, NF-κB-CFP and AP-1-mCherry) to generate triple parameter reporter (TPR) cells. A selected TPR clone derived from these cells was analyzed for reporter gene expression (open histograms: unstimulated; grey histograms: PMA/Ionomycin-treated). Numbers indicate geometric fluorescence intensity (gMFI). (**B**) J76 TPR were equipped with CD8 and tumor (PRAME, BOB1) or virus (CMV) antigen-specific T cell receptors (TCRs). Expression analysis of the reporter genes, CD3, CD8 and the T cell receptors was performed by flow cytometry. Numbers indicate percentage (upper) and gMFI (lower). (**C**) Activation kinetics of NFAT, NF-κB and AP-1 when stimulating J76 TPR PRAME with JY-LCL cells loaded with 1 μM PRAME peptide at the indicated timepoints. Histograms of unstimulated (open) and stimulated (filled) reporters are depicted on the right side. Numbers indicate percentage of positive reporters.

To generate a cellular platform for analysis of TCRs, our previously described transcriptional reporter constructs for NF-κB, NFAT and AP-1 were introduced into the J76 cell line [16]. This cell line is a derivative of the human T cell lymphoma line Jurkat E6.1 and is characterized by the lack of expression of the TCR alpha and beta chains [9]. The resulting J76 TPR were screened with phorbol 12-myristate 13-acetate (PMA) and Ionomycin and a single cell clone showing high induction of all three reporter genes (NFAT-eGFP, NF-κB-CFP and AP-1-mCherry) was selected for further use (Figure 1A). Three TCRs previously cloned in our laboratory were selected for evaluation in these reporter cells: a TCR specific for a well characterized cytomegalovirus antigen (CMV) pp65 peptide (NLVPMVATV; HLA-A*02:01 restricted; [17]) and TCRs specific for peptides derived from the tumor antigens preferentially expressed antigen in melanoma (PRAME; SLLQHLIGL; HLA-A*02:01 restricted; [18]) and B cell-specific octamer binding protein-1 (BOB1; APAPTAVVL; HLA-B*07:02 restricted; [19]). The J76 TPR were co-transduced to express these TCRs as well as CD8 and subsequently flow-sorted for expression of these molecules. The resulting cell lines co-expressed CD3 and CD8 and stained positive with a pan TCR αβ-antibody (Figure 1B). To find the optimal timepoint for reporter activation and to compare the kinetics of NFAT, NF-κB and AP-1 a time course stimulation experiment was performed. J76 TPR PRAME cells were stimulated with the EBV transformed B cell line JY-LCL loaded with 1 uM PRAME peptide and reporter activity was measured at 8, 12, 24 and 48 h. All three transcription factors reached their maximum of positive reporters after 24 h (Figure 1C). Hence, J76 based reporter cells with three different TCRs could be successfully generated for the simultaneous analysis of NFAT, NF-κB and AP-1 activation after 24 h.

### Functional evaluation of the antigen-specific J76 TPR

**Figure 2:**
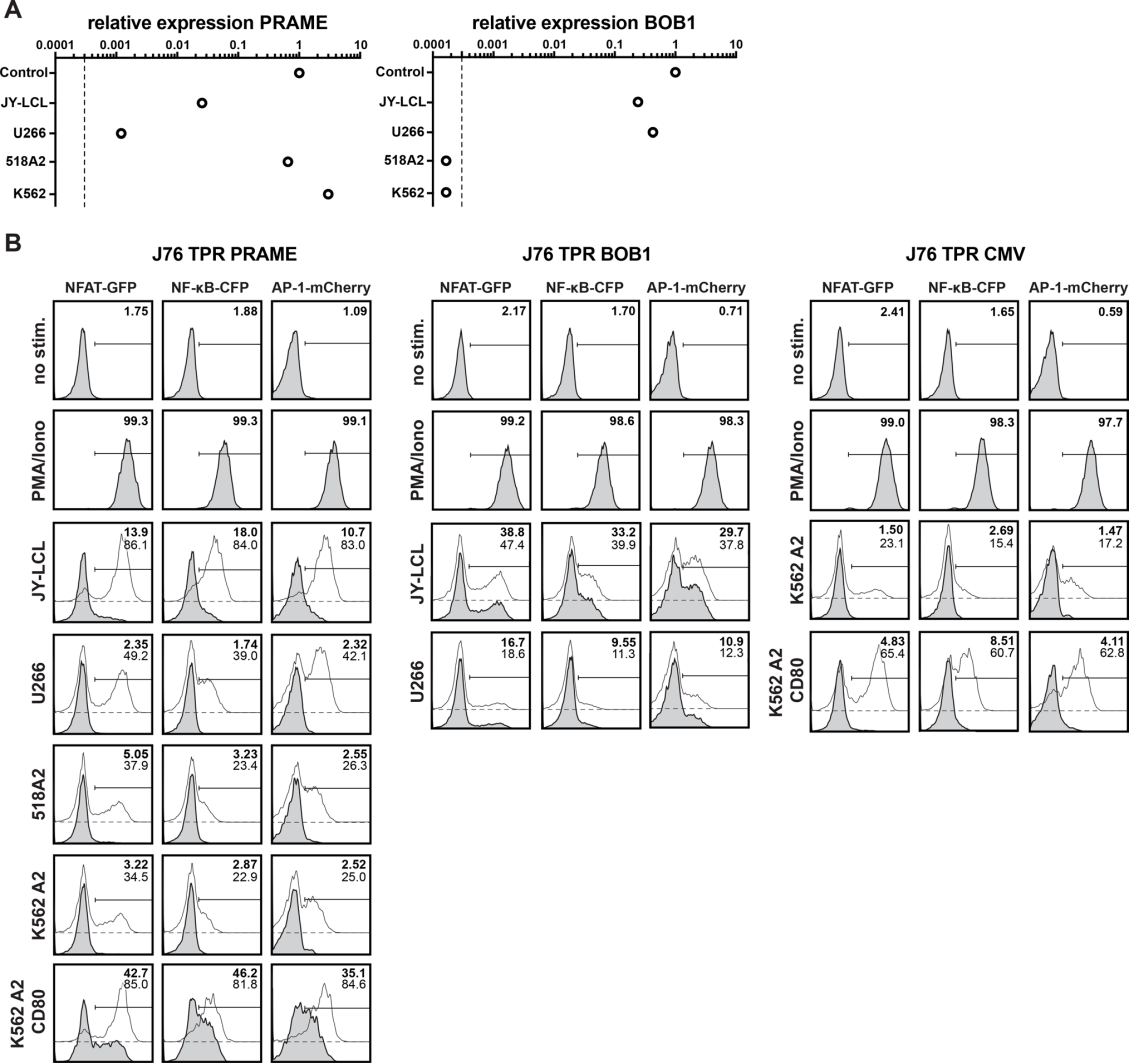
Functional evaluation of the antigen-specific J76 TPR (**A**) Expression of PRAME and BOB1 mRNA in different cell lines as quantified by qPCR. The dashed line indicates the detection limit. (**B**) Different tumor and melanoma cell lines were used as antigen presenting cells to stimulate J76 TPR PRAME (left), BOB1 (middle) or and CMV (right). In addition to analysis of endogenous peptide presentation (grey histograms), 1 uM of antigenic peptides was added to the cocultures (open histograms). Numbers show the percentage of positive cells of unloaded (bold) or peptide loaded (thin) assay conditions. Depicted histograms are representative for three independently performed experiments.

To assess antigen specific responses of the TCR-tg reporters we performed coculture experiments with different cancer cell lines. Relative expression of PRAME or BOB1 in these cancer cell lines was analyzed by qPCR (Figure 2A). Reporter assays showed that J76 TPR PRAME cells specifically responded to cells presenting PRAME peptide in the context of HLA-A2^+^ (Figure 2B left panel). Addition of exogenous peptide increased reporter activation as expected. However, it was noted that the response to endogenously processed PRAME presented by different cell lines did not correlate with the expression levels measured by qPCR. 518A2 cells for instance, which expressed high levels of PRAME failed to induce significant reporter activation, whereas JY-LCL cells induced strong responses despite low PRAME expression. Moreover, it was observed that expression of CD80 in K562-A2^+^ cells strongly enhanced their capacity to activate PRAME specific reporter cells. This prompted us to analyze expression of costimulatory molecules on cancer cell lines and we observed that JY-LCL cells expressed high levels of the CD28 ligands CD80 and CD86, whereas these molecules were not detected on 518A2, K562 and U266 cells, which only elicited weak responses (Supplementary Figure 1A). Higher activation of reporter cells expressing a TCR specific for BOB1 to JY-LCL cells compared to U266 cells is also likely due to expression of CD28 ligands by JY-LCL cells. Loading with additional BOB1 peptide did only marginally enhance reporter activity which is in line with previous findings [19] (Figure 2B middle panel, Supplementary Figure 1A). CMV TCR-tg reporter cells were cocultured with K562-A2^+^ and K562-A2^+^-CD80^+^ cells. Since K562 cells do not express CMV-antigens, reporter responses were only induced upon addition of antigenic peptides (Figure 2B right panel). Again the presence of CD80 on K562-A2^+^ led to greatly enhanced reporter responses. Collectively, these results demonstrate specific responses of TCR-transgenic J76 reporters to their respective antigens, and they also highlight that costimulatory signals strongly enhance antigen specific responses of our reporter cells.

### Introduction of adhesion molecules enhances TPR sensitivity

**Figure 3:**
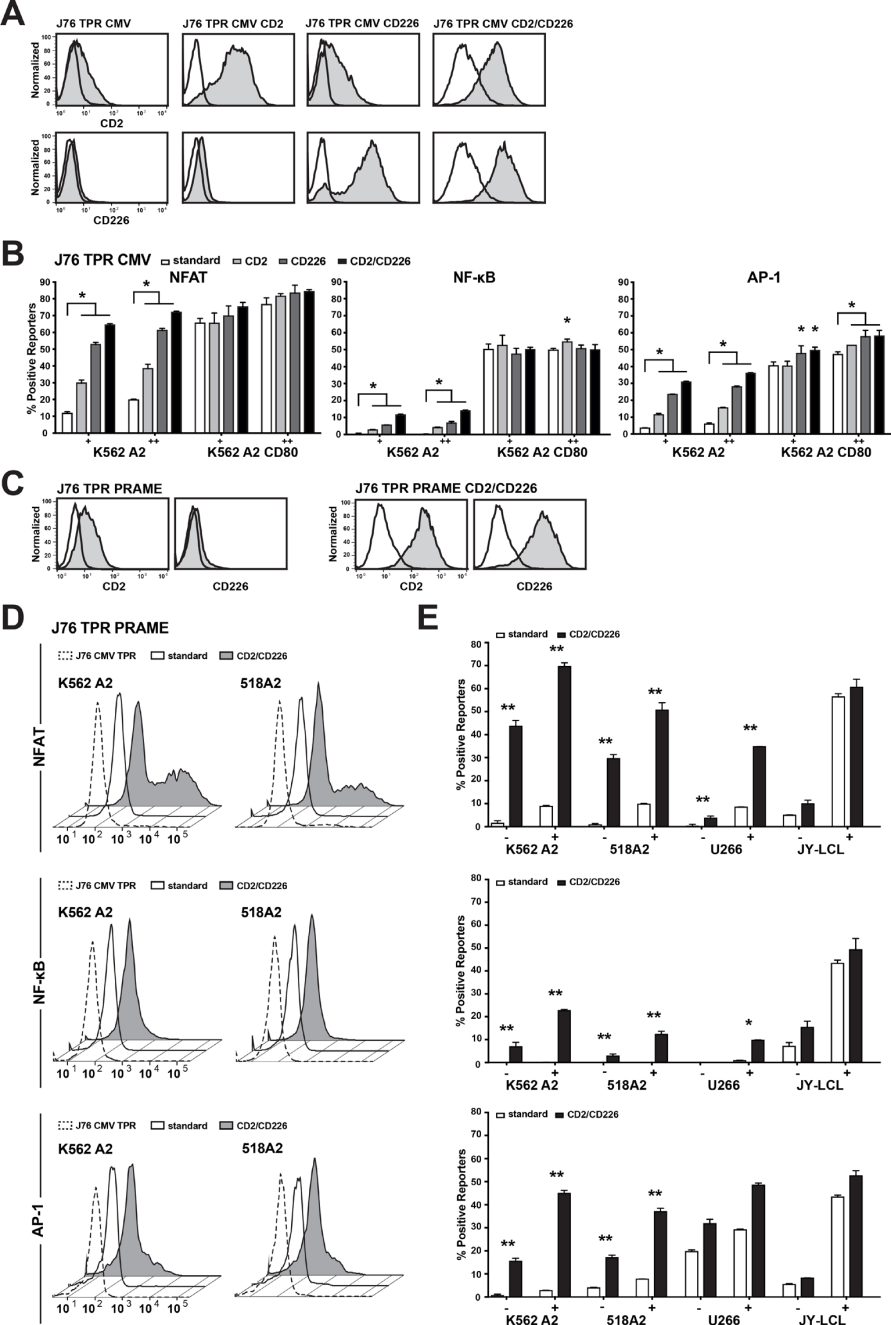
Introduction of adhesion molecules enhances TPR sensitivity (**A**) The adhesion molecules CD2 and CD226 were expressed on the J76 TPR CMV individually or together and were evaluated by flow cytometry. Open histograms show the appropriate isotype control. (**B**) Engineered APCs (eAPCs) were loaded with 100 nM (+) or 1 uM (++) pp65 peptide and were used to evaluate the individual effect of the adhesion molecules on the J76 CMV TPR sensitvity. (**C**) CD2 and CD226 were expressed on J76 TPR PRAME and their expression was tested by flow cytometry. Open histograms show the appropriate isotype control. (**D**, **E**) J76 PRAME TPR (Standard) and J76 PRAME TPR CD2/CD226 were used to screen different tumor and melanoma cell lines for response to endogenous PRAME presentation. (D) Histograms show recognition of endogenous PRAME antigen presentation by the different TPRs. J76 TPR CMV CD2/CD226 were used as a negative control. (E) Bar diagrams show responses to endogenous PRAME (-) and cells loaded with 100 nM PRAME peptide (+), respectively. Mean percent of positive reporters is shown for duplicate values and experiment shown is representative of three independent experiments. Statistical analysis was performed using Wilcoxon (B) and Friedman (E) tests (^*^P ≤ 0.05, ^**^P ≤ 0.01, ^***^P ≤ 0.001).

It is well established that numerous receptor ligand interactions promote T cell activation in addition to CD28-CD80/CD86. Therefore, we compared the widely used Jurkat line E6.1 (JE6.1) and Jurkat 76 (J76) cells regarding the expression of accessory receptors implicated in T cell activation processes. Pronounced differences were seen for the activating receptors CD2 and CD226, which potently promote the activation of primary human T cell as well as T cell lines [20–23]. J76 cells were negative for CD226 and expressed low levels of CD2, whereas high levels of both molecules were detected on the JE6.1 subline (Supplementary Figure 1B). Since it was observed that CD58, the ligand for CD2, and CD112 and CD155, which function as ligands for CD226, are ubiquitously expressed in different cancer cell lines (Supplementary Figure 1A), we investigated whether introducing these molecules in our TCR-transgenic reporters will increase their sensitivity for their cognate antigens. In a first step, we used the J76 CMV TPR to overexpress CD2 or CD226 or both molecules (Figure 3A) and tested the reporter reactivity with the K562 HLA-A2^+^ and K562 HLA-A2^+^CD80^+^ cells loaded with antigenic peptide (Figure 3B). Both accessory molecules significantly increased the reporter activation and an additive effect was seen when using the CD2^+^CD226^+^ J76 CMV reporter. Introduction of CD2/CD226 had a stronger effect on the induction of NFAT compared to NF-kB reporters. Importantly, the impact of these molecules was more pronounced upon stimulation with K562-A2^+^ cells compared to stimulation with K562-A2^+^-CD80^+^ cells indicating that in absence of CD28 signaling accessory signals are especially important (Figure 3B). Next, we generated J76 PRAME TPR expressing high levels of CD2 and CD226 (Figure 3C). These reporters had strongly enhanced sensitivity to the PRAME antigen when compared to the PRAME-specific TPR not over-expressing CD2/CD226. While it was not possible to detect PRAME-specific responses against the K562 HLA-A2^+^ cells or the 518A2 melanoma cells using the standard reporter, the CD2^+^CD226^+^ J76 PRAME TPR were stimulated by endogenously processed PRAME antigen expressed by these cells (Figure 3D). Again, the overexpression of CD2 and CD226 had most pronounced effects on reporter cells stimulated by cells not providing CD28 ligands, therefore only modest differences were observed in the response to JY-LCL, which expressed significant levels of CD80/CD86 (Figure 3E). Hence, CD2^+^CD226^+^ overexpression greatly increases the sensitivity of TCR-tg J76 reporters to their cognate antigens in the absence of CD28 stimulation.

### Chimeric CD28 receptors boost sensitivity to antigen

**Figure 4:**
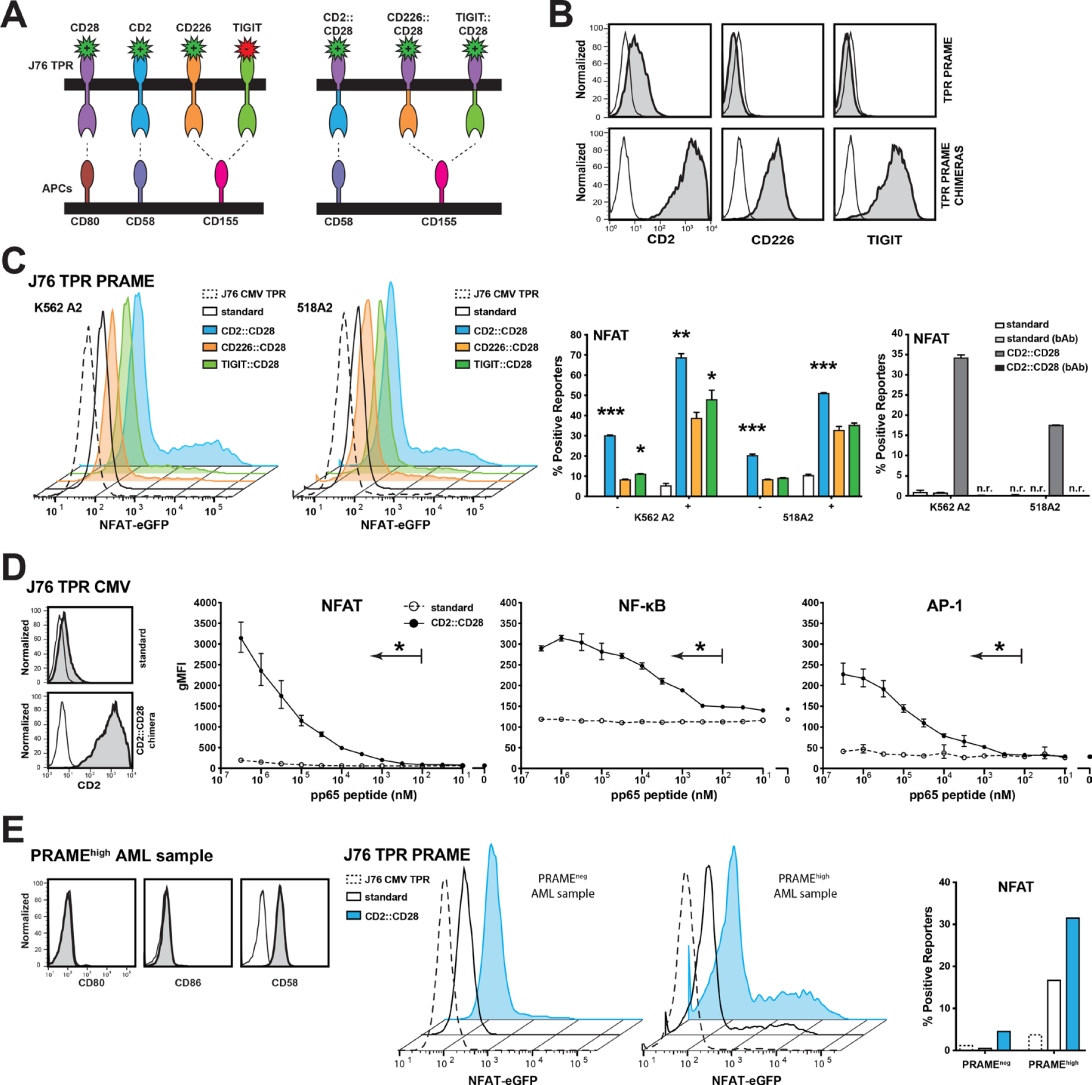
Chimeric CD28 receptors boost TPR sensitivity (**A**) Schematic illustration of the generated chimeric CD28 receptors. (**B**) Expression analysis of the chimeric CD28 receptors (grey) or appropriate isotype control (open) on J76 TPR PRAME using flow cytometry. (**C**) Unloaded (–) or 100 nM peptide loaded (+) K562-based engineered APCs (eAPC) and 518A2 melanoma cells were used to evaluate the potential of the chimeric CD28 receptors. Depicted histograms show NFAT activation of different PRAME TPRs by endogenous PRAME antigen presentation. J76 TPR CMV CD2::CD28 is shown as negative control. Color of histograms and bars correspond to colors of chimeric receptors depicted in (A). Right panel: A CD58 blocking antibody (bAb; 10 μg/mL) was used to confirm the specific contribution of the CD2::CD28 chimera; n.r. no reactivity. (**D**) J76 CMV TPR were equipped with the CD2::CD28 chimera (left). The sensitivity of the resulting reporter and the standard CMV reporter to stimulation with K562 HLA-A2^+^ cells loaded with antigenic peptide at different concentrations was determined (right). Geometric mean flourescent intensity of reporters is shown for duplicate values and experiment is representative of three independent experiments. (**E**) A primary AML sample that showed high PRAME expresssion was tested for expression of CD80, CD86 and CD58 (left). J76 CMV TPR (negative control), J76 PRAME TPR and J76 PRAME TPR CD2::CD28 were cocultured with PRAME^neg^ and PRAME^high^ AML samples and NFAT reporter responses are depicted (right). Statistical analysis was performed using Wilcoxon (C) and Friedman (D) tests. Differences to standard reporters are shown (^*^P ≤ 0.05, ^**^P ≤ 0.01, ^***^P ≤ 0.001).

It is well established that the primary costimulatory signal CD28 has an essential role in the induction of productive immune responses [24]. Ankri *et al.* have recently demonstrated that a chimeric PD-1 molecule comprising of the extracellular domain of PD-1 fused to intracellular CD28 sequences provides T cells that interact with target cells expressing PD-1-ligands with costimulatory signals [25]. We aimed to assess whether chimeric CD28 molecules have utility to enhance the response of our TCR-tg reporter cells towards their cognate antigens. The broad expression of CD58, CD112 and CD155 on cancer cells provided a rationale to assess CD2::CD28 and CD226::CD28 chimeras. CD112 and CD155 also serve as binding partners for the inhibitory receptor T cell immunoreceptor with Ig and ITIM domains (TIGIT) (Figure 4A) [26]. Since TIGIT has a higher affinity for these ligands than CD226 [27], we also generated TIGIT::CD28 chimeras. J76 PRAME TPR were transduced with the chimeric constructs (Figure 4B) and then functionally evaluated for endogenous PRAME recognition using K562 HLA-A2^+^ and 518A2 cells. All three molecules enhanced the reporter sensitivity, however the best reporter induction was detected using the CD2::CD28 chimeric receptor, which strongly responded to antigenic peptide processed from endogenously expressed PRAME. A CD58 blocking antibody abrogated enhanced responses of reporters expressing the CD2::CD28 chimeric receptor (Figure 4C). Experiments where we stimulated CMV specific J76 TPR cells with K562 cells loaded with different concentrations of antigenic peptide revealed that expression of CD2::CD28 increased the sensitivity of the reporters more than thousand fold (Figure 4D). We evaluated the response of J76 PRAME TPR expressing CD2::CD28 receptors to primary acute myeloid leukemia (AML) cells that express no CD28 ligands CD80 and CD86 (Figure 4E). These experiments revealed that reporters expressing CD2::CD28 chimeric receptors showed greatly enhanced response to AML cells expressing PRAME. Taken together, our results indicate that introducing receptors that induce CD28 signals upon encounter of TCR-tg T cells with their target cells greatly improves their response.

## DISCUSSION

Adoptive T cell therapy has been shown to be an effective strategy to combat cancer but also life-threatening virus infections following hematopoietic stem cell transplantation [28–31]. Compared to classical strategies based on the administration of *ex vivo* expanded naturally occurring specific T cells, the use of genetically engineered T cells offers several advantages. It allows to select the most suitable antigens and to target them with TCRs selected for exquisite specificity and affinity [32]. Whereas the endogenous repertoire may lack TCRs strongly reacting with relevant tumor antigen presented in the context of self-MHC molecules, several studies have demonstrated that such high affinity TCRs can be isolated from individuals that do not express the relevant restriction element [18, 19, 33–35]. Extensive characterization of candidate TCRs for adoptive T cell therapy is mandatory to increase the therapeutic efficacy and reduce the risk of adverse events. Primary TCR-transgenic T cells are indispensable for this purpose but their generation is work-intense and associated with several obstacles including variations between T cells prepared from different donors and miss-pairing with the endogenous TCR chains. More robust, immortalized cellular systems for the evaluation of TCRs would thus be desirable to complement studies with primary T cells. Jurkat 76 is a human T cell line devoid of alpha and beta TCR chains and these cells thus have been extensively used to study function and specificity of conventional alpha/beta TCRs but also of TCRs derived from MAIT cells or δ/αβ T cells [7, 36, 37]. However, reading out the activation of J76 has proven to be difficult because the cells show only limited upregulation of activation markers and cytokine production. Here, we describe the generation and use of a triple parameter reporter Jurkat 76 cell line to evaluate tumor and virus antigen-specific human TCRs. Our reporter cells allow to simultaneously assess the activity of the transcription factors NF-κB, NFAT and AP-1, which play a major role in T cell activation processes. We generated reporter lines expressing three previously cloned human TCRs specific for peptides derived from the tumor antigens PRAME (SLLQHLIGL; HLA-A*02:01 restricted; [18]) and BOB1/p44 (APAPTAVVL; HLA-B*07:02 restricted; [19]) and to a well-characterized virus antigen CMV pp65 peptide (NLVPMVATV; HLA-A*02:01 restricted; [17]). While PRAME (preferentially expressed antigen on melanomas) shows only minute expression in healthy tissues, high expression was found in diverse cancers like acute or chronic lymphoblastic or myeloid leukemia, making it an interesting target for T cell-based therapy [38]. The intracellular B cell-specific transcription factor BOB1 is abundantly presented in B cell malignancies including acute lymphoblastic leukemia, chronic lymphocytic leukemia and mantle-cell lymphoma but also in multiple myeloma [19, 39–42]. The pp65 protein of CMV (cytomegalovirus capsid derived antigen) is a major target of immune responses in CMV infections [43].

The Jurkat based TCR-transgenic reporter cells specifically respond to their cognate antigens presented by tumor cell lines or engineered antigen presenting cells in a dose-dependent manner. Several studies have shown that protein antigen expression does not inevitably correlate with pMHC density [44, 45]. In this context, our reporter cells can be used as an indicator how reprogrammed T cells will recognize potential tumor target cells. In addition, they are a potent, robust and cost-effective tool for side-by-side analysis of different TCRs obtained from natural repertoires and evaluation of TCRs engineered for increased affinity [44–46]. Importantly, TCR-transgenic reporter cells integrate antigen-specific and accessory signals. Therefore, strategies to increase the responses of adoptively transferred T cells to tumor cells can be readily assessed. We found that CD58 and CD155, which function as binding partners for the adhesion molecules CD2 and CD226, respectively, are broadly expressed in cell lines derived from human melanomas or B cell tumors. Overexpressing CD2 and CD226 on our T cell reporters greatly increased their sensitivity to tumor cells presenting their cognate antigens.

CD28 can be regarded as the primary costimulatory receptor and lack of CD28 signals might limit the success of adoptive therapy with TCR-tg T cells. To overcome this problem, Ankri *et al.* and others have designed so-called switch receptors that comprise for example the extracellular domain of PD-1 fused to intracellular CD28 sequences and sustain CD28 signaling in the immune-suppressive tumor microenvironment [25, 47, 48]. We have adopted this strategy and devised chimeric receptors where CD28-signaling domains were fused to the extracellular binding domains of either CD2, CD226 or TIGIT and thus can be engaged by CD58 or CD155 expressed on the tumor targets. We found that all tested chimeric receptors were effective but differed in their efficiency. Of note, fusion with the extracellular domain of CD2 showed the highest potential to introduce CD28 signaling. Hence, combining melanoma antigen-specific TCRs with CD2::CD28 chimeric receptors might have potential in adoptive T cell therapy, since melanoma cells were shown to have high expression of CD58 [49]. Leukemia cells like AML cells are also CD58 positive and we demonstrate greatly enhanced recognition of primary AML cells by PRAME-specific J76 reporters equipped with CD2::CD28 [50].

Genetic manipulation of T cells offers a plethora of options to improve their responses to target cells [2]. Depending on the tumor targets such chimeric receptors could bind different ligands and in addition they could introduce distinct costimulatory signals. Importantly, such signals vary considerably in their quality as demonstrated for CD28 and 4-1BB in respect of NFAT activation [16]. Our data suggest that introduction of CD2/CD226 signals induces strong NFAT but weaker NF-κB activation. Since our reporters read out the activity of three major transcription factors, they will allow to gain insight on the strength as well as the quality of signals induced upon interaction of engineered T cells with their target cells. TCR-tg reporter cells could be instrumental to assess chimeric receptors targeting different tumor-expressed molecules thereby helping to develop improved adoptive T cell therapies.

This study is the first to present an antigen specific reporter system for the evaluation of TCR signaling via NF-κB, NFAT and AP-1 using the Jurkat 76 T cell line. The triple parameter reporters can be easily employed for cost-efficient and quick analysis of cloned TCRs as well as other antigen receptor formats like chimeric antigen receptors (CARs) and modifications thereof. The presentation of the cognate antigenic peptide on tumor cells or virus-infected cells can be readily assessed with these cells. This system is useful to complement research on primary T cells in comparing various strategies to increase responses of engineered T cells to target cells.

## MATERIALS AND METHODS

### Cell culture, antibodies and flow cytometry

The human Jurkat T cell line derivative Jurkat 76 and the human cell lines used as APC, K562, a human erythroleukemia cell line (ATCC^®^ CCL-243), U266, a human B lymphocytic multiple myeloma cell line (ATCC^®^ TIB-196), 518A2, a human melanoma cell line transduced to express HLA-A2 [18], and the EBV transformed B cell lines JY-LCL were cultured using IMDM containing 10% heat-inactivated FBS, 100 μg/mL streptomycin and 100 U/mL penicillin in a humidified atmosphere (5% CO_2_) at 37° C. PRAME SLL peptide (SLLQHLIGL), CMV pp65 peptide (NLVPMVATV) and BOB1 p44 peptide (APAPTAVVL) were produced at the Leiden University Medical Center (LUMC, Leiden). The following monoclonal antibodies were used to confirm surface expression on the Jurkat triple parameter reporter cell lines: CD3-PE-Cy7 (UCHT1, Biolegend), CD8β-PE (2J/8.5H7, Beckmann-Coulter), TCRαβ-FITC (WT31, BD Bioscience), TCRαβ-APC (IP26, Biolegend), CD28-APC (CD28.2, Biolegend), CD2-PE (TS1/8, Biolegend), CD226-PE (11A8, Biolegend) and appropriate isotype controls. A purified functional grade CD58 (TS2/9; LEAF™) antibody was used for blocking studies (Biolegend). Flow cytometry analysis was performed using FACSCalibur™, LSRFortessa™ and LSR II™ flow cytometers (BD Bioscience). FlowJo software (version 10.0.6., Tree Star, Ashland, OR) was used for data analysis. Overlayed histograms are normalized so that each curve is scaled to 100%. Peripheral blood was obtained from patients suffering from acute myeloid leukemia after informed consent, and procedures with human material were performed in accordance with the Helsinki Declaration of 1975.

### Expression constructs and retroviral/lentiviral transduction

The expression construct encoding CD8 was cloned into the retroviral vector pLZRS, and packaging cells Φ-NX-A were used for viral supernatant generation [51, 52]. cDNAs of CD2 and CD226 were cloned into the retroviral vector pCJK2 [21]. Constructs encoding chimeric receptors harboring the cytoplasmic domains of CD28 were generated by fusing sequences encoding the extracellular domain of CD2 (UniProt P06729, aa 1-209), CD226 (UniProt Q15762, aa 1-254) or T cell immunoreceptor with Ig and ITIM domains (TIGIT; UniProt Q495A1, aa 1-141) with the transmembrane and cytoplasmic domains of CD28 (UniProt P10747, aa 153-220). The cDNAs were cloned into the lentiviral vector pHR harboring a puromycin selection marker [53]. DNA sequencing was performed to confirm the integrity of the resulting expression constructs.

### Generation of antigen-specific triple parameter T cell reporters

The TCR αβ negative Jurkat 76 cell line was transduced with previously described reporter constructs NFAT-eGFP, NF-κB-CFP and AP-1-mCherry [16, 54]. Established single cell clones were tested for low background expression of the reporter genes and strong upregulation of eGFP, CFP and mCherry upon stimulation with 5 ng/mL PMA and 400 ng/mL Ionomycin (both from Sigma Aldrich). The CD8 coreceptor was retrovirally introduced and CD8^+^ cells were selected. The α-chain and β-chain of the TCRs specific for the epitopes PRAME, CMV and BOB1 were retrovirally expressed in the resulting TPR cells using the pMP71 vector [9, 17, 55]. The CMV-TCR is specific for the CMV-pp65 antigen (NLVPMVATV; restricted to HLA-A*02:01; [17]), the PRAME-TCR is specific for the SLL peptide derived from the tumor antigen PRAME (SLLQHLIGL; restricted to HLA-A*02:01; [18]) and the BOB1-TCR is specific for the B cell transcription factor BOB1 (APAPTAVVL; restricted to HLA-B*07:02; [19]). Ultimately, single cell clones were established (limiting dilution) and were selected based on their capability for specific TCR triggering. To increase the sensitivity of the reporters accessory receptors (CD2, CD226) or chimeric CD28 receptors (CD2::CD28, CD226::CD28, TIGIT::CD28) were overexpressed and the resulting bulk reporters were sorted for high expression of the molecules.

### Cocultivation experiments of tumor cell lines or primary AML samples with antigen-specific TPR

Tumor cell lines (3 × 10^5^/well, labeled with the fluorescent cell membrane labeling dye PKH26 from Sigma Aldrich, PKH26GL-1KT) were treated with peptides (100 nM or 1 μM) or mock-treated and cocultured with the antigen-specific TPR lines (5 × 10^5^/well) in 96-well round bottom plates in a final volume of 100 μL. The term “exogenous loading” refers to the addition of peptides to the cocultures throughout the manuscript. Stimulation with PMA and Ionomycin was used as positive control. After 24 h, cells were harvested and reporter gene expressions were measured using a FACSFortessa™ flow cytometer (BD Biosciences). Tumor cell lines (labeled with PKH26) were excluded from the analysis based on their fluorescence. In addition, PRAME negative and positive primary AML samples were thawed, rested overnight in IMDM at 37° C, stained with PKH26 and tested for specific antigen recognition with J76 PRAME and CMV TPR using an E:T ratio of 1:5. For each sample reporter gene induction is expressed as percent positive reporters with negative reporters gated based on J76 cells unstimulated to calculate median and standard deviation from duplicate or triplicate wells.

### Quantitative real-time PCR of PRAME and BOB1

Expression of PRAME and BOB1 was analyzed using qPCR. Isolation of total RNA from the APCs was performed using the RNAqueous™-mico kit (Ambion, Life Technologies) according to the manufacturer’s instruction. M-MLV reverse transcriptase (Invitrogen) was used to generate cDNA and gene expression was analyzed on the Roche Lightcycler 480 (Roche, Basel, Switzerland) with EvaGreen^®^ dye (Biotium, Hayward, California) and FastStart™-TaqDNA Polymerase (Roche). For PRAME and BOB1 detection the following primer pairs were used: forward primer 5′ CGTTTGTGGGGTTCCATTC 3′ together with reverse primer 5′ GCTCCCTGGGCAGCAAC 3′ (PRAME)[18]; forward primer 5′-TGTCACGACAAGAAGCTCCG-3′ together with reverse primer 5′- GTGGGTAGTGTGGAAAGGGG-3′ (BOB1) [19]. Expression levels measured in the melanoma cell line Mel1.14 (PRAME) and CD19^+^ B cells derived from peripheral blood of healthy individuals (BOB1, mean value) were set to 1.

### Statistics

Statistical analysis was performed with GraphPad Prism (version 7, GraphPad So ware, Inc., La Jolla, CA). Two-sided paired non-parametric *t*-tests for two (Wilcoxon test) or more groups (Friedman test followed by Dunn’s multiple comparison test) were conducted (^*^P ≤ 0.05, ^**^P ≤ 0.01, ^***^P ≤ 0.001).

## SUPPLEMENTARY MATERIALS FIGURE



## Abbreviations

AML: acute myeloid leukemia
AP-1: activator protein-1
BOB1: B cell-specific octamer binding protein-1
CMV: cytomegalovirus
eAPC: engineered antigen presenting cell
gMFI: geometric mean fluorescence intensity
JE6.1: Jurkat E6.1
J76: Jurkat 76
JY-LCL: lymphoblastoid cell lines
NFAT: nuclear factor of activated T cells
NF-κB: nuclear factor kappa-light-chain-enhancer of activated B-cells
PMA: phorbol 12-myristate 13-acetate
PRAME: preferentially expressed antigen in melanoma
TCR-tg: T cell receptor-transgenic
TIGIT: T cell immunoreceptor with Ig and ITIM domains
TPR: triple parameter reporter
