# Supplementary material for: A Jurkat 76 based triple parameter reporter system to evaluate TCR functions and adoptive T cell strategies
Source: Oncotarget. 2018 Apr 3;9(25):17608–19. doi: 10.18632/oncotarget.24807 (PMC5915142; doi:10.18632/oncotarget.24807)
Supplement: Supplementary file 1 [file oncotarget-09-17608-s001.pdf]

# A Jurkat 76 based triple parameter reporter system to evaluate TCR functions and adoptive T cell strategies

## SUPPLEMENTARY MATERIALS

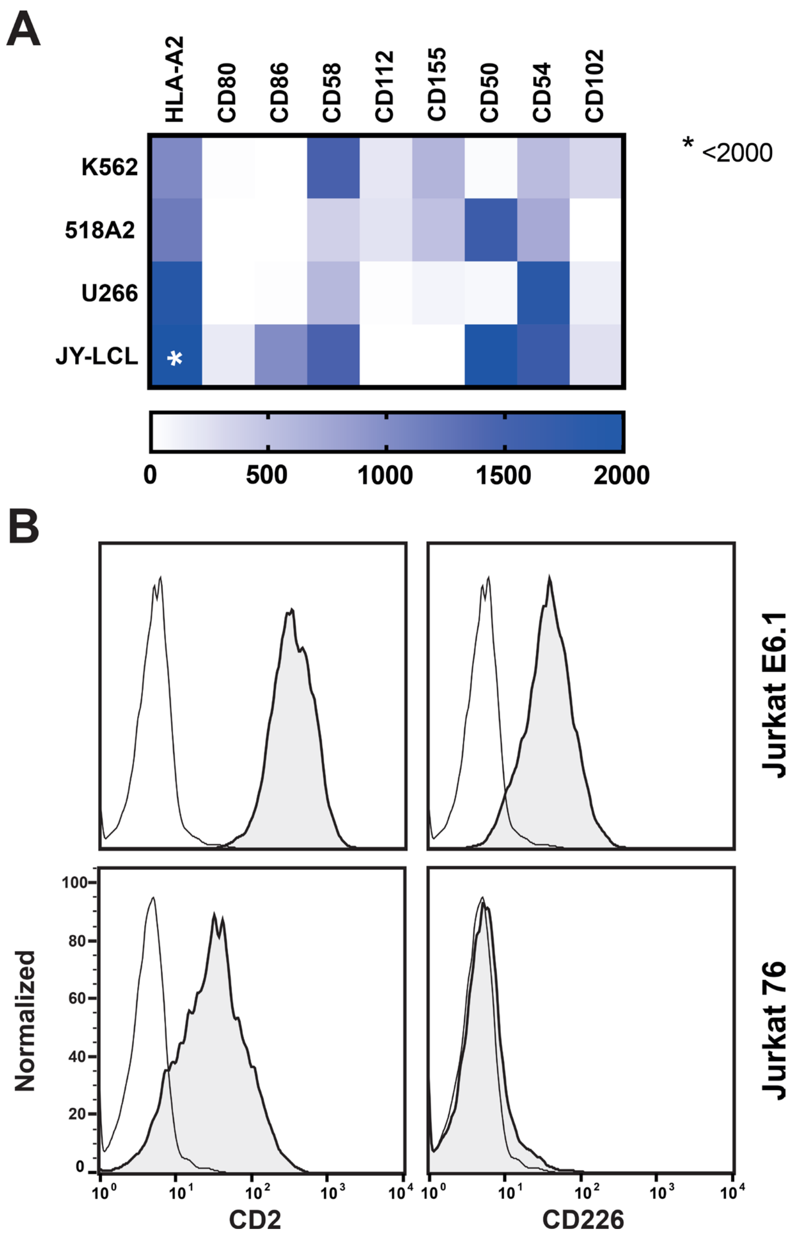


**Supplementary Figure 1: Characterization of tumor cell lines and Jurkat T cell lines.** (**A**) Expression of costimulatory and adhesion moleules was measured by flow cytometry and geometric mean flourescent intensity (gMFI) is shown in the form of a heatmap. (**B**) The two Jurkat T cell lines JE6.1 and J76 were analyzed for their expression of the adhesion molecules CD2 and CD226.
